# Moving low value care lists into action: prioritizing candidate health technologies for reassessment using administrative data

**DOI:** 10.1186/s12913-018-3459-1

**Published:** 2018-08-15

**Authors:** Lesley J. J. Soril, Brayan V. Seixas, Craig Mitton, Stirling Bryan, Fiona M. Clement

**Affiliations:** 1 Department of Community Health Sciences, Cumming School of Medicine, University of Calgary, Calgary, AB Canada; 2 Health Technology Assessment Unit, O’Brien Institute for Public Health, University of Calgary, Calgary, AB Canada; 3 Centre for Clinical Epidemiology and Evaluation, Vancouver Coastal Health Research Institute, University of British Columbia, Vancouver, BC Canada; 4 School of Population and Public Health, University of British Columbia, Vancouver, BC Canada; 5 British Columbia SUPPORT Unit, Vancouver, BC Canada

**Keywords:** Health technology reassessment, Low value care, Disinvestment, Administrative health data, de-adoption, de-implementation

## Abstract

**Background:**

Active management of existing health technologies (e.g., devices, diagnostic, and/or medical procedures) to ensure the delivery of high value care is increasingly recognized around the world. A number of initiatives have raised awareness of technologies that may be overused, mis-used, or potentially harmful by compiling them into lists of low value care. However, despite the growing number of lists, changes to local healthcare practices remain challenging for many systems. The objective of this study was to develop and implement a process, leveraging existing initiatives and data assets, to produce a list of prioritized low value technologies for health technology reassessment (HTR).

**Methods:**

An expert advisory committee comprised of clinical experts and health system decision-makers was convened to determine key process requirements. Once developed, the process was piloted to assess feasibility in the Canadian province of British Columbia (BC).

**Results:**

The expert advisory committee identified five required attributes for the process: data-driven, routine and replicable, actionable, stakeholder collaboration, and high return on investment. Guided by these attributes, a 5-step process was developed. First, over 1300 published low value technologies (i.e., from the National Institute for Health and Care Excellence [NICE] “do not do” recommendations, low value technologies in the Australian Medical Benefits Schedule, and Choosing Wisely “Top 5” lists) were identified. Using appropriate coding systems for BC’s administrative health data (e.g., International Classification of Diseases [ICD]), the low value technologies were queried to examine frequencies and costs of technology use. This information was used to rank potential candidates for reassessment based on high annual budgetary impact. Lastly, clinical experts reviewed the ranked technologies prior to broad dissemination and stakeholder action. Pilot testing of the process in BC resulted in the prioritization of 9 initial candidate technologies for reassessment.

**Conclusions:**

This is the first account of a systematic approach to move a collective body of low value technology recommendations into action in a healthcare setting. This work demonstrates the feasibility and strength of using administrative data to identify and prioritize low value technologies for HTR at a population-level.

**Electronic supplementary material:**

The online version of this article (10.1186/s12913-018-3459-1) contains supplementary material, which is available to authorized users.

## Background

Given the scarcity of resources and financial pressures that exist for all public health systems, the need for active management of existing health services to ensure delivery of high value care is increasing [1–5]. Particular attention has been placed on mechanisms to reassess services and procedures currently in the health system that may be unnecessary or of low value for certain patient groups or circumstances [6–9]. This can include the overuse or misuse of ineffective, inefficient, or even potentially harmful technologies [10].

Consequently, a number of international efforts have focused on raising awareness of low value care by developing lists of such technologies. Among the most notable of these “list-making” initiatives include the United Kingdom’s National Institute for Care and Health Excellence (NICE) “Do Not Do” recommendations [11], the list of over 150 low value technologies in the Australian Medical Benefits Schedule (MBS) [12], and the international Choosing Wisely campaign’s “Top 5” lists of low value tests and procedures [13]. By considering current evidence of effectiveness and/or efficiency, these collective sources in total comprise over a thousand recommendations of low value technologies currently used in practice [11–13].

However, despite this extensive collection of recommendations, understanding their relevancy (i.e., quantifying the extent of low value care) and acting to change such practice has been challenging for local health systems. In particular, challenges are thought to arise due to the lack and/or infancy of reliable administrative processes for identifying and prioritizing these technologies [14]. In the absence of such guidance the uptake of low value technology recommendations and change in clinical practice has historically been passive and slow [14].

Interestingly, increasing access to and use of routinely collected administrative health data assets have led to major advancements in quantifying the effects of low value care at the population-level [15–18]. For example, regional variations in practice across Canada were examined using administrative health data and up to 30% of test orders, treatments, and procedures associated with 8 of the Choosing Wisely Canada recommendations, were identified to be of low value [19]. The analytical capacity of routinely collected data, coupled with the already extensive collection of published low value care recommendations, may therefore offer substantial opportunity to systematize the identification and prioritization of low value technologies for reassessment, and potential disinvestment, for local health system contexts.

The objective of this study was to develop a data-driven methodological process, leveraging existing knowledge and data assets, to develop a list of prioritized candidate technologies for health technology reassessment (HTR). HTR is defined as the structured, evidence-based evaluation of the medical, economic, social, and ethical impacts of a technology currently used in the system, in order to inform its optimal use compared to its alternatives [20, 21]. A case example of the implementation of the data-driven process to advance HTR initiatives within a Canadian health system context will also be described.

## Methods

### Expert stakeholder engagement

Based on recommended best practices for the conduct of HTR initiatives [21], an expert advisory committee composed of 14 clinical experts and health system decision-makers was convened to provide timely feedback on process features in order to, ultimately, increase the likelihood of process buy-in and uptake. All of the committee members were identified based on their role as champions within the health system and/or their past experiences in advisory capacities or direct involvement in previous health technology assessment (HTA) processes and, as such, were considered crucial stakeholders for the implementation and sustainability of HTR initiatives. The primary responsibilities of the committee were to provide critical feedback on the outputs of the research team and recommend key process requirements for the selection of candidate technologies for reassessment. The research team—composed of academic researchers in health economics, health technology assessment, priority setting, and health policy—served as process stewards, facilitating expert advisory committee meetings and conducting all evidence syntheses and analytical tasks.

### Evidence synthesis

The research team completed a rapid review of the published literature to identify any applied and/or theoretical approaches to HTR, particularly for the identification and prioritization of technologies for reassessment. A recent, high-quality scoping review previously examined the literature in this area to March 2014 [22]. The scoping review was considered the most comprehensive account of the literature to date and was updated for the current rapid review. Briefly, an electronic database search of literature published from January 2014 to July 2016 was conducted. Guided by the search strategy of the scoping review, terms such as *reassessment*, *disinvestment*, *de-adoption* were searched in MEDLINE, PubMED, EMBASE, Cochrane Database of Systematic Reviews, Cochrane Central Register of Controlled Trials, and CINAHL. Identified citations were reviewed by two independent reviewers using the inclusion and exclusion criteria presented in Additional file 1: Appendix 1.

To supplement the information collected from the rapid review, an environmental scan of the grey literature was performed. The websites of established HTA organizations were searched to identify relevant reports, presentations, guidelines, working papers, or other pertinent grey literature. Relevant organizations were identified from a systematic review [23] and experiential knowledge of the research team. A complete list of organizations is provided in Additional file 2: Appendix 2. Similar terms used to search the published literature were applied. From both the rapid review and environmental scan, a total of 18 references (e.g., published articles, reports, and websites) were included for final analysis.

### Expert consensus process

The findings of the rapid review and environmental scan were presented at a half-day in-person meeting to the expert advisory committee for review and deliberation. The research team compiled common mechanisms to identify and prioritize low value technologies from the literature, including use of local data sources to identify aberrant patterns in technology use (e.g., temporal, geographic and/or provider variations), regular surveillance of new clinical and cost-effectiveness evidence for existing technologies, and nominations of candidate technologies from local clinical and health system experts [24]. The expert advisory committee was asked to consider the relevancy of such findings with existing decision-making mechanisms and reach consensus on key process requirements for the selection of candidate technologies for reassessment.

## Results

### Attributes and overview of the technology selection process

**Fig. 1:**
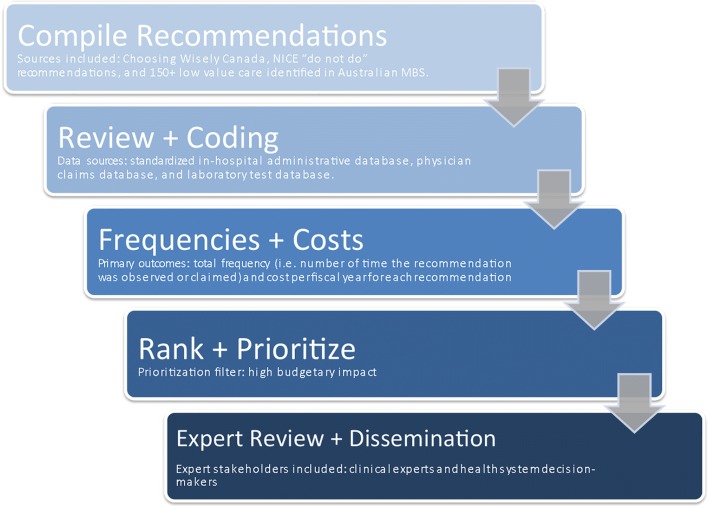
Methodological Process for Selecting Candidate Technologies for HTR. Guided by the process attributes recommended by the expert advisory committee, a 5-step methodological process was developed. First, published low value technologies from the NICE “do not do” recommendations, low value technologies in the Australian MBS, and Choosing Wisely Canada lists were compiled. Secondly, the low value recommendations were reviewed and coded using the appropriate coding systems for the administrative health data. In the third and fourth steps, low value technologies were queried in the administrative data to examine frequencies and costs of technology use, and this information was subsequently used to rank potential candidates for HTR based on high annual budgetary impact. Lastly, clinical experts reviewed the ranked technologies prior to broad dissemination and stakeholder action

Through the consensus exercise, the expert advisory committee agreed that the methodological process must be: data-driven; routine and replicable; actionable; enable stakeholder collaboration; and ultimately, yield a high return on investment. Guided by these process requirements, the research team then devised a 5-step methodological process for the selection of candidate technologies for HTR (Fig. 1). The details for each step are outlined below.

### Compilation of existing low value care recommendations

Low value care recommendations were compiled from the NICE “Do Not Do” recommendations [11], the list of over 150 low value technologies in the Australian MBS [12], and from the Canadian Choosing Wisely lists of low value tests and procedures [25] as of July 27th, 2016. Each technology was listed in a recommendation because it was identified to be of limited or no clinical benefit for select population. A description of each of these sources is provided in Table 1.

**Table 1:** Overview of Sources of Low Value Care Recommendations

National Institute for Health and Care Excellence (NICE) Do Not Do Recommendations	
As part of their technology assessment infrastructure in the United Kingdom, NICE has evaluated the clinical- and cost-effectiveness of existing technologies concomitant to the assessment of new technologies in a process known as multiple technology assessment (MTA). MTAs involve evidence syntheses of clinical- and cost-effectiveness of a given technology and its alternatives, as well as guidance for policy or practice implementation. Over time, a number of low values technologies currently in use in the National Health Service have been identified. Referred to as the “Do Not Do” recommendations, NICE has developed the most extensive collection of existing technologies of uncertain effect or absence of evidence.	
Low value technologies in the Australian Medical Benefits Schedule (MBS)	
Elshaug et al. [12] identified over 150 candidate technologies for reassessment in the Australian MBS. The Australian government funded the study to implement an evidence-based process to manage the MBS and ensure continued listing of clinical- and cost-effective technologies. A multiplatform approach was used to survey the peer-reviewed literature, conduct a targeted database search of reassessment recommendations in other jurisdictions, and obtain expert input through sampling of local stakeholders.	
The International Choosing Wisely Campaign	
Choosing Wisely is one of the most widely implemented low value list-making initiatives. Founded in the United States by the American Board of Internal Medicine, and now in over 12 countries, the Choosing Wisely campaign engaged medical societies to develop lists of low value tests and procedures across various medical specializations. Processes to develop the lists have varied from expert consensus exercises to systematic and non-systematic reviews of the literature. The campaign is intended to facilitate conversations between physicians and patients about unnecessary tests and treatments and make smart and effective choices to ensure high-quality care. Specifically in Canada, there are over 30 lists and over 200 low value tests and procedures.	

### Review and coding of recommendations in appropriate coding systems

Data from 3 administrative health databases were examined for use of low value technologies: a standardized hospital database of the administrative aspects of care for all hospital admissions (e.g., diagnostic information, health services administered, billing information); a physician claims database of services and procedures claimed for reimbursement; and a laboratory database of all tests ordered and reimbursed. The databases were selected based on the following considerations: capture of the relevant patient cohorts; timely access of the database; and coverage of use of/claims for non-drug technologies in hospital and by physicians which could be easily identified with administrative data coding language. Specifically, in the hospital administrative data, patient conditions or diagnoses are coded using the International Statistical Classification of Diseases and Related Health Problems, 10th Revision, Canada or ICD-10-CA system [26], and in-hospital services and procedures are coded using the Canadian Classification of Health Interventions or CCI system [27]. For the physician claims and laboratory data, patient conditions are coded using the ICD-9-CM system [28], and the services or procedures for reimbursement are coded using database-specific billing or fee item codes [29, 30].

In the low value care recommendations, a technology was either listed (e.g., discectomy) or use of a technology was described as low value for a given clinical indication or diagnosis (e.g., computerized tomography or ultrasound to diagnose appendicitis). To examine use of these technologies in administrative data, the technology and clinical indications in the recommendations were mapped to the appropriate ICD and/or fee codes for the respective databases. A recommendation was excluded from the coding process if it: referred to a technology whose coverage fell outside of the purview of the decision-making or reviewing body (e.g., a drug technology where the remit of the committee is exclusively non-drug technologies); was not publicly-funded; contained a technology with no identifiable service/procedural, billing, or fee item code; or contained language or qualifiers that could not be identified in the administrative data (i.e., language was clinically nuanced). If a recommendation specified characteristics identifiable within the data sources (i.e., age range and/or gender), such restrictions were also added to the coded recommendations. Included recommendations were coded in duplicate and a third reviewer was consulted to ensure an accurate and exhaustive collection of codes. All of the coding was tracked in Microsoft Excel.

### Analysis of frequencies and attribution of costs to administrative data

Use of the low value technologies in the final coded recommendations were then examined in the in-hospital and claims health data between April 1, 2010 and March 31, 2015. The anonymized, aggregate-level administrative data sources were queried for the following outcomes: the total frequency (i.e., number of time the recommendation was observed or claimed) and cost per fiscal year for each recommendation. Annual costs from physician and laboratory claims are parameters directly available from the respective claims databases. For the in-hospital data, annual costs were calculated using the case-mix grouper (CMG)-costing methodology developed in Canada to enable gross-costing estimates [31]. Briefly, the average CMG-derived resource intensity weight (RIW) from the in-hospital data, an estimate of the average per patient in-hospital costs, was multiplied by: (1) the cost per standard hospital stay (CSHS) for BC [32]; and (2) the total frequency for a given fiscal year examined.

### Ranking and prioritization of technologies based on high budgetary impact

To prioritize candidate technologies for HTR, a *high budgetary impact* filter was applied to the coded recommendations that resulted in any observed use (i.e., frequency and cost from any of the administrative health databases). High budgetary impact was defined as total estimated expenditures (i.e., in-hospital and claims) exceeding 1 million Canadian dollars ($1 M) in any fiscal year of the 5-year period. This budgetary impact may result from high cost per technology use, high volume or use, or an aggregate measure of both. If a coded recommendation resulted in zero frequency or cost it was assumed that the low value technology was not used during the study period.

### Expert advisory committee review and dissemination

The draft list of prioritized candidate technologies for HTR, filtered and ranked based on high budgetary impact, was presented and discussed with the expert advisory committee in one meeting, and in ad hoc one-on-one meetings with clinical stakeholders of the committee. Input from these stakeholders is required to ensure relevancy and feasibility of a HTR for each of the prioritized candidate technologies (i.e., pattern of use and/or cost was worth examining). The input received from the expert advisory committee was documented in a report that accompanied the prioritized list of technologies. These documents were then disseminated broadly to relevant health system stakeholders for consideration.

### Case example: Pilot testing in British Columbia

The Canadian province of British Columbia (BC) was used as the case example to pilot test the technology selection process. In BC, the provincial Ministry of Health is responsible for overseeing the legislation and regulations of health services available for the over 4.6 million BC residents [33]. The BC Ministry of Health also manages several provincial programs and services including the Medical Services Plan (MSP), which covers most physician-based services. Health technologies considered for funding in the BC healthcare system are vetted through the BC Ministry of Health’s Health Technology Assessment Committee (HTAC). The scope of the HTAC includes the assessment of new non-drug health technologies (e.g., devices, diagnostic, and/or medical procedures) and the reassessment of technologies already embedded within the health system. This provided an ideal, collaborative opportunity in which to pilot test the proposed data-driven process in a real-world health system context.

**Fig. 2:**
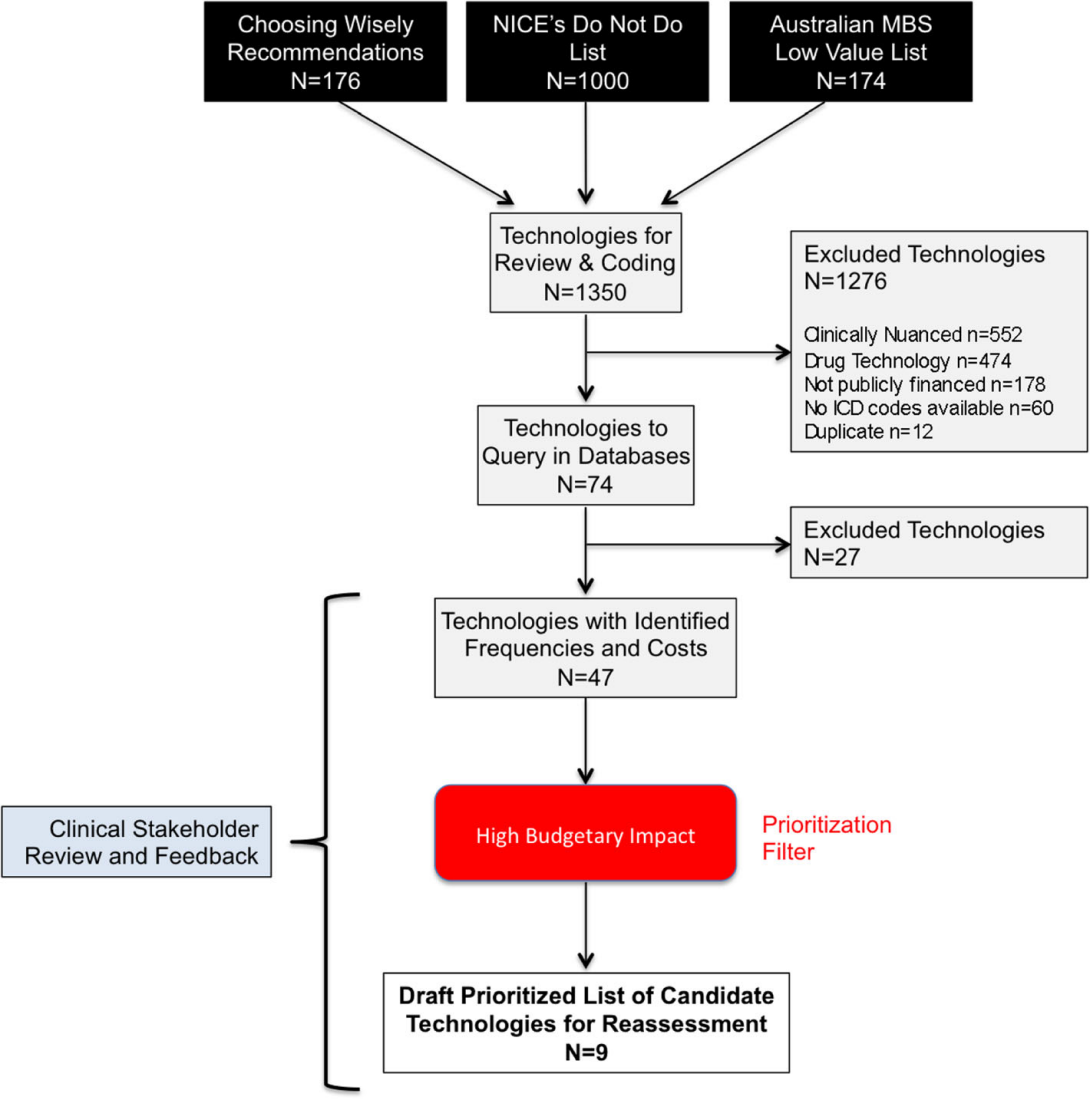
Flow Chart from the Pilot Testing. A total of 1350 low value technologies were reviewed from the three source lists. Twelve-hundred and seventy-six were excluded because the language in the recommendation was clinically nuanced (*n* = 552), it referred to drug technologies (*n* = 474), technologies were not publicly-funded in the BC health system (*n* = 178), had no identifiable service/procedural, billing or fee item code (*n* = 60), or were duplicates (*n* = 12). Seventy-four low value technologies were coded and, of these, 47 were found to have frequencies and costs between April 1, 2010 and March 31, 2015. Nine potential candidate technologies were prioritized based on high budgetary impact (costs > $1 M in a fiscal year). The expert advisory committee, particularly the clinical stakeholders, reviewed the technologies with identified frequencies and costs and provided feedback on the prioritized candidate technologies to ensure relevancy and feasibility for HTR

A schematic of the steps and resultant number of technologies from pilot testing the process are outlined in Fig. 2. A total of 1350 low value technology recommendations were consolidated and reviewed from the three source lists [11, 12, 25]. A considerable number of recommendations (*n* = 552) contained language or qualifiers that could not be identified in the administrative data (i.e., recommendation was found to be clinically nuanced) and were therefore excluded. Examples of this exclusion criterion are provided in Table 2. Technologies not publicly-funded within the BC health system (*n* = 178), such as complementary and alternative treatments, were excluded from the coding process. Many of the recommendations referring to drug technologies (*n* = 474) were also excluded as public drug coverage falls outside of the decision-making purview of the HTAC. In addition, for 60 recommendations no identifiable service/procedural, billing or fee item code could be identified for the technology. The greatest overlap was observed between the Australian MBS list and the NICE do not do recommendations. Ultimately, 1276 low value technology recommendations were excluded.

**Table 2:** Examples of “Clinically Nuanced” Language Not Identifiable in Administrative Data

Language from low value care recommendations	Example reasons for exclusion
• “low-risk patients” • “high-risk patients” • “unless directly indicated by the risk profile of the patient”	Imprecise/unexplained consideration of risk.
• “must not be used to prevent” • “should not be used as a diagnostic tool” • “should not be used as first-line treatment”	Unable to determine rationale underlying physicians’ decision for use of technology.
• “asymptomatic patients” • “absence of clinical indications” • “with uncomplicated symptoms” • “unless red flags are present” • “without alarm symptoms”	Qualifying words are vague; did not mention any specific signal or symptom.

Seventy-four low value technology recommendations were coded and of these, 47 were observed in at least one of the administrative health databases (i.e., any frequency and cost) between April 1, 2010 and March 31, 2015. When the total in-hospital, physician, and laboratory claims expenditures were prioritized according to the high budgetary impact filter (i.e., costs greater than $1 M in a fiscal year), a total of 9 potential candidate technologies were prioritized for HTR. These technologies primarily came from the list of 150 low value technologies on the Australian MBS list and the NICE “do not do” recommendations. The majority (*n* = 6) were used in-hospital (i.e., identified in the hospital database) and, of these, three were concomitantly identified within the physician claims data. The 3 remaining technologies were identified only in the physicians and laboratory claims data.

## Discussion

To our knowledge, this is the first account of a systematic approach to move a collective body of low value technology recommendations into action. Established international lists of low value recommendations—drawn from reviews of the literature [12], deliberative expert stakeholder processes [25], and multiple health technology assessments [11]—form the foundation of the methodological process. Administrative data was used to (1) examine the extent to which low value care exists within a health system and (2) rationally prioritize technologies for HTR based on budgetary impact. From the pilot test, a total of 9 candidate technologies of demonstrable high budgetary impact were prioritized. These technologies serve as targets to improve the quality and appropriateness of care, while collectively also representing a substantial opportunity for disinvestment.

The need to identify and prioritize low value technologies is consistently described as the critical, and often difficult, first phase in a reassessment process [22, 24, 34–36]. Other proposed frameworks have described ‘triggers’ to identify and prioritize low value technologies, including publication of new high-quality evidence, nomination by local stakeholders, identified variations in technology use, significant volume or estimated cost savings, and recommendations against use by an external body [24, 37]. However, while these frameworks offer key process requirements (which have been considered in this present work), they do not provide insight into how and/or where one would begin the process of selecting a technology. It is unclear if the triggers should be considered in a specific order, or if considered equal, or what mechanisms need to be in place to investigate them. In our 5-step process, we sought to address this uncertainty. Our starting collection of established, international low value recommendations also serves as an efficient beginning as they have been previously considered and filtered based on clinical outcomes.

The list of 9 candidate technologies also demonstrates the feasibility and strength of using administrative data to locally identify and prioritize technologies for reassessment. We were able to establish the extent to which the low value technology recommendation may or may not exist within the BC health system, while concomitantly understanding the associated budgetary impact. It is important to note that the 9 prioritized technologies in this study are merely examples of those that may undergo reassessment. There are additional areas for potential reassessment, as indicated by the observed frequency and cost data for 38 other low value recommendations in our search. Thus, it is possible for other technologies to be prioritized with the use of an alternate prioritization filter.

Engagement of the expert advisory committee from the outset of the study was a foundational component to the data-driven process [21]. The combined expertise and leadership roles of committee members within the clinical, operational, and policy domains of the health system ensured relevancy of the prioritized candidate technologies and their reassessment in the BC healthcare context. With that said, when the expert advisory committee completed an initial review of the technologies no changes to the prioritized candidate list was made. Following the subsequent targeted clinical stakeholder review, additional technologies were also suggested for inclusion as potential future reassessment topics. Such suggestions were documented and put forward to the BC Ministry of Health.

One of the greatest challenges in the review and coding step was the exclusion of the substantial number of low value recommendations from the process. The finely nuanced language observed in several recommendations, wherein specification of certain populations and/or clinical contexts could not be ascertained within administrative data, resulted in the most exclusions (*n* = 552). Essentially, the simpler the phrasing, the more likely a recommendation would be coded and queried with the administrative data. For other health systems contexts, linking administrative data with other available clinical databases (e.g., clinical registries) that collect more detailed clinical information, or having direct involvement of clinical experts to review and code the recommendations, may help to overcome the challenges experienced in the present case example. In addition, developers of future low value care lists may consider tapering use of nuanced language in order to produce more explicit recommendations that can be appropriately applied to coded administrative data assets.

While our present work leveraged local administrative data to prioritize technologies for reassessment from a large breadth of potential candidates, use of such data to examine utilization patterns of select low value technologies have been described. One of the first accounts was in Australia, for example with ophthalmology services, where administrative data was used to identify geographic and socioeconomic variations in claims and to examine temporal and economic trends over time [38]. In Canada, significant, practice-level variability in the rates of low-value bone density scans, back pain imaging, and cervical cancer screening was identified through administrative data in the province of Ontario [39]. Further, administrative data has been used to evaluate the impact of Choosing Wisely recommendations on clinical practice [16, 19, 40]. In the United States, for example, trends in the use of low value tests and procedures before and after the Choosing Wisely lists were evaluated using health insurance claims data [17, 41, 42]. Across these studies, patterns in use post-recommendation were found to be inconsistent, with use in some technologies decreasing, others even increasing, and some whose use remained completely unchanged.

### Limitations and future considerations

There are a number of limitations and challenges encountered during this work worth noting. First, recommendations referring to low value drug technologies comprised a large number of those excluded (*n* = 474). For this present work, such technologies were not coded simply because public drug coverage falls outside of the scope of decision-making for the BC Ministry of Health’s HTAC. However, in health systems with administrative databases and coding systems for publicly-funded prescription drugs, the systematic process may be similarly applied to identify and prioritize candidate drug technologies for reassessment.

It should also be noted that two of the sources of recommendations are continually adding to their lists [11, 25] and other new collections of low value care continue to be developed [43–45]. Therefore, the present data-driven process should be continually updated as clinical practice evolves and new low value recommendations become published. In addition, there was limited clinical input beyond members of the advisory committee. Technology nominations were not formally solicited beyond those prioritized directly from the administrative data. Future consultation exercises, particularly with clinical experts specializing in the areas of the excluded “clinically nuanced” recommendations, should be conducted. It is highly likely that a broader call for opportunities for reassessment to additional clinical groups and specialty areas will identify further priority areas for consideration.

With regards to the analysis of the administrative data, issues such as coding error by the reviewers and exclusion of technologies that are not tracked within the administrative databases (i.e., codes not available) may have underestimated the extent and costs of low value care in our case example. In contrast, our estimated in-hospital costs do not solely reflect use of the technology, but are rather based on the resource intensity weights that factor in the total costs accrued by an average patient that received that service or procedure with that technology. As such, all of the in-hospital costs overestimate the total burden incurred from the use of low-value technology in the hospital.

Due to the anonymized, aggregate-level nature of the administrative data, we were unable to comment on geographic nor provider variations in technology use, both of which are previously described as characteristic of low value technology use [24]. Further, the case example of the data-driven process specifically sought to explore the advent and patterns of low value technology use (i.e., overuse and/or misuse) and not underuse of high value care. To ensure optimal use of all technologies in the healthcare system, future work employing a parallel data-driven process, instead using existing high value technology recommendations, should be explored.

Lastly, it is important to acknowledge that the identification and prioritization of candidate technologies is simply the first step in the reassessment process. Once a candidate(s) is selected further evidence (e.g., contextual issues around use of the technology) must be collected and considered to form a policy decision on optimal use, as well as strategies to implement the decision [21]. Such strategies will depend on the technology, the health system, and the stakeholders involved in making and impacted by the decision [46] and can range from de-listing a technology from publicly-funded medical benefits to implementation of behaviour change interventions, such as audit and feedback, targeting healthcare provider practices [47]. While the process presented in this work aids to quantify the extent and costs of low value technologies, qualitative methodology to understand the facilitators and/or barriers to changing low value care practice might prove more useful for developing tailored strategies and interventions within a given health system [48, 49].

## Conclusions

Diffusion of low value technology recommendations into clinical practice has typically been passive in nature, with no formal or structured guidance on how to identify the existence or extent of such low value care. The proposed process provides a systematic mechanism for prioritizing candidate technologies that may be recommended for reassessment and has been demonstrated in the case example of the BC provincial health system. This process acknowledges that evidence to indicate that a technology is of low value should be substantiated from not only an empirical stance, but a local one as well (i.e., evidence of local use, support from local clinical stakeholders). This work is a critical step for all HTR programmes and is a significant contribution toward the technology management agenda in Canada and internationally.

## Additional files

Additional file 1: Appendix 1. Inclusion and Exclusion Criteria for the Rapid Review of the Published Literature. The inclusion and exclusion criteria that were applied when reviewing the identified citations during the rapid review. (DOCX 31 kb)

Additional file 2: Appendix 2. Organizations and Agencies Examined for the Environmental Scan. A list of HTA organizations that were searched to identify relevant reports, presentations, guidelines, working papers, or other pertinent grey literature as part of the environmental scan. (DOCX 31 kb)

## Abbreviations

BC: British Columbia
CCI: Canadian Classification of Health Interventions
CMG: Case Mix Grouper
CSHS: Cost per Standard Hospital Stay
HTA: Health Technology Assessment
HTAC: Health Technology Assessment Committee
HTR: Health Technology Reassessment
ICD: International Classification of Diseases
MBS: Medical Benefits Schedule
MSP: Medical Services Plan
NICE: National Institute for Health and Care Excellence
RIW: Resource Intensity Weight

## References

[1] Saini V, Brownlee S, Elshaug AG, Glasziou P, Heath I (2017). Addressing overuse and underuse around the world. Lancet.

[2] Saini V, Garcia-Armesto S, Klemperer D, Paris V, Elshaug AG, Brownlee S, Ioannidis JP, Fisher ES (2017). Drivers of poor medical care. Lancet.

[3] Elshaug AG, Rosenthal MB, Lavis JN, Brownlee S, Schmidt H, Nagpal S, Littlejohns P, Srivastava D, Tunis S, Saini V (2017). Levers for addressing medical underuse and overuse: achieving high-value health care. Lancet.

[4] Brownlee S, Chalkidou K, Doust J, Elshaug AG, Glasziou P, Heath I, Nagpal S, Saini V, Srivastava D, Chalmers K (2017). Evidence for overuse of medical services around the world. Lancet.

[5] Glasziou P, Straus S, Brownlee S, Trevena L, Dans L, Guyatt G, Elshaug AG, Janett R, Saini V (2017). Evidence for underuse of effective medical services around the world. Lancet.

[6] Glasziou P, Moynihan R, Richards T, Godlee F. Too much medicine; too little care. 2013, 347:f4247. 10.1136/bmj.f4247 23820022

[7] Levinson W, Kallewaard M, Bhatia RS, Wolfson D, Shortt S, Kerr EA (2015). ‘Choosing wisely’: a growing international campaign. BMJ Qual Saf.

[8] Volpp KG, Loewenstein G, Asch DA (2012). Choosing wisely: low-value services, utilization, and patient cost sharing. JAMA.

[9] Garner S, Littlejohns P (2011). Disinvestment from low value clinical interventions: NICEly done?. BMJ.

[10] Elshaug AG, McWilliams JM, Landon BE (2013). The value of low-value lists. JAMA.

[11] Do Not Do Recommendations [https://www.nice.org.uk/savingsandproductivity/collection?page=1&pagesize=2000&type=donotdo]. 2016. Accessed 27 Jul 2016.

[12] Elshaug AG, Watt AM, Mundy L, Willis CD (2012). Over 150 potentially low-value health care practices: an Australian study. Med J Aust.

[13] The Choosing Wisely Lists [http://www.choosingwisely.org/getting-started/lists/]. 2017. Accessed 13 June 2016.

[14] Elshaug AG, Hiller JE, Tunis SR, Moss JR (2007). Challenges in Australian policy processes for disinvestment from existing, ineffective health care practices. Aust N Z Health Policy.

[15] Kelman CW, Pearson S, Day RO, Holman CDJ, Kliewer EV, Henry DA (2007). Evaluating medicines: let's use all the evidence. Med J Aust.

[16] Bhatia RS, Levinson W, Shortt S, Pendrith C, Fric-Shamji E, Kallewaard M, Peul W, Veillard J, Elshaug A, Forde I (2015). Measuring the effect of choosing wisely: an integrated framework to assess campaign impact on low-value care. BMJ Quality & Safety.

[17] Schwartz AL, Landon BE, Elshaug AG, Chernew ME, McWilliams JM (2014). Measuring low-value care in Medicare. JAMA Intern Med.

[18] Collins B (2016). Big data and health economics: strengths, weaknesses, opportunities and threats. PharmacoEconomics.

[19] Canadian Institute for Health Information: Unnecessary Care in Canada. (CIHI ed. Ottawa, ON; 2017.

[20] Noseworthy T, Clement F (2012). Health technology reassessment: scope, methodology, & language. Int J Technol Assess Health Care.

[21] Soril LJ, MacKean G, Noseworthy TW, Leggett LE, Clement FM (2017). Achieving optimal technology use: a proposed model for health technology reassessment. SAGE Open Med.

[22] Niven DJ, Mrklas KJ, Holodinsky JK, Straus SE, Hemmelgarn BR, Jeffs LP, Stelfox HT (2015). Towards understanding the de-adoption of low-value clinical practices: a scoping review. [review]. BMC Med.

[23] Leggett LE, Mackean G, Noseworthy TW, Sutherland L, Clement F (2012). Current status of health technology reassessment of non-drug technologies: survey and key informant interviews. Health Res Policy Systems.

[24] Elshaug AG, Moss JR, Littlejohns P, Karnon J, Merlin TL, Hiller JE (2009). Identifying existing health care services that do not provide value for money. Med J Aust.

[25] The Lists [http://www.choosingwiselycanada.org/recommendations/]. 2016. Accessed 27 Jul 2016.

[26] International Statistical Classification of Diseases and Related Health Problems, Tenth Revision, Canada, Volume One — Tabular List [https://www.cihi.ca/en/icd_volume_one_2015_en.pdf]. 2015. Accessed 15 Aug 2016.

[27] Canadian Classification of Health Interventions, Volume Three — Tabular List [https://www.cihi.ca/en/cci_volume_three_2015_en.pdf]. 2015. Accessed 15 Aug 2016.

[28] Diagnostic Code Descriptions (ICD-9) [http://www2.gov.bc.ca/gov/content/health/practitioner-professional-resources/msp/physicians/diagnostic-code-descriptions-icd-9]. 2016. Accessed 15 Aug 2016.

[29] MSC Payment Schedule [http://www2.gov.bc.ca/assets/gov/health/practitioner-pro/medical-services-plan/msc-payment-schedule-june-2016.pdf]. 2016. Accessed 15 Aug 2016.

[30] Schedule of Fees For the Laboratory Services Outpatient: Payment Schedule [https://www2.gov.bc.ca/assets/gov/health/practitioner-pro/laboratory-services/laboratory_services_schedule_of_fees.pdf]. 2015. Accessed 15 Aug 2016.

[31] Canadian Institute for Health Information: Acute Care Grouping Methodologies: From Diagnosis Related Groups to Case Mix Groupers Redevelopment. Ottawa; 2004.

[32] Your Health System: Cost of a Standard Hospital Stay 2016. Accessed 4 Aug 2016.

[33] Ministry of Health [http://www2.gov.bc.ca/gov/content/governments/organizational-structure/ministries-organizations/ministries/health]. 2017. Accessed 26 Apr 2017.

[34] (Osteba) BOfHTA: Report on the development of the GUNFT guideline: Quality plan for the NHS MHSP. . (Reports HTA ed., vol. No 2007/11 2009; 2009.

[35] MacKean G, Noseworthy T, Elshaug AG, Leggett L, Littlejohns P, Berezanski J, Clement F (2013). Health technology reassessment: the art of the possible. Int J Technol Assess Health Care.

[36] Seo H-J, Park JJ, Lee SH (2016). A systematic review on current status of health technology reassessment: insights for South Korea. Health Res Policy Systems.

[37] Paprica PA, Culyer AJ, Elshaug AG, Peffer J, Sandoval GA (2015). From talk to action: policy stakeholders, appropriateness, and selective disinvestment. Int J Technol Assess Health Care.

[38] Merlin T SJ, Holton C, Mundy L, Tamblyn D, Ellery B, Juneja V, Reddin E, Scrimgeour S, Hennessy S,: review of MBS items for specific ophthalmology services under the MBS quality framework. Canberra, ACT: Commonwealth of Australia; 2011.

[39] Pendrith C, Bhatia M, Ivers NM, Mecredy G, Tu K, Hawker GA, Jaglal SB, Wilson L, Wintemute K, Glazier RH (2017). Frequency of and variation in low-value care in primary care: a retrospective cohort study. CMAJ Open.

[40] Brett J, Elshaug AG, Bhatia RS, Chalmers K, Badgery-Parker T, Pearson S-A (2017). A methodological protocol for selecting and quantifying low-value prescribing practices in routinely collected data: an Australian case study. Implement Sci.

[41] Rosenberg A, Agiro A, Gottlieb M, Barron J, Brady P, Liu Y, Li C, DeVries A (2015). Early trends among seven recommendations from the choosing wisely campaign. JAMA Intern Med.

[42] Hong AS, Ross-Degnan D, Zhang F, Wharam JF (2017). Small decline in low-value back imaging associated with the ‘choosing Wisely’Campaign, 2012–14. Health Aff.

[43] Soon J, Buchbinder R, Close J, Hill C, Allan S, Turnour C (2016). Identifying low-value care: the Royal Australasian College of Physicians' EVOLVE initiative. Med J Aust.

[44] Less Is More Collection [https://jamanetwork.com/collections/44045/less-is-more]. 2017. Accessed 17 May 2017.

[45] Too Much Medicine [http://www.bmj.com/too-much-medicine]. 2017. Accessed 17 May 2017.

[46] Soril LJJ, Niven DJ, Esmail R, Noseworthy TW, Clement FM (2018). Untangling, unbundling, and moving forward: Framing health technology reassessment in the changing conceptual landscape. Int J Technol Assess Health Care.

[47] Colla CH, Mainor AJ, Hargreaves C, Sequist T, Morden N (2017). Interventions aimed at reducing use of low-value health services: a systematic review. Med Care Res Rev.

[48] Colquhoun HL, Squires JE, Kolehmainen N, Fraser C, Grimshaw JM (2017). Methods for designing interventions to change healthcare professionals’ behaviour: a systematic review. Implement Sci.

[49] Michie S, Johnston M, Abraham C, Lawton R, Parker D, Walker A (2005). Making psychological theory useful for implementing evidence based practice: a consensus approach. Qual Saf Health Care.

