# Supplementary material for: Moving low value care lists into action: prioritizing candidate health technologies for reassessment using administrative data
Source: BMC Health Serv Res. 2018 Aug 15;18:640. doi: 10.1186/s12913-018-3459-1 (PMC6094474; doi:10.1186/s12913-018-3459-1)
Supplement: Supplementary file 1 — Appendix 1. Inclusion and Exclusion Criteria for the Rapid Review of the Published Literature. The inclusion and exclusion criteria that were applied when reviewing the identified citations during the rapid review. (DOCX 31 kb) [file 12913_2018_3459_MOESM1_ESM.docx]

**Appendix 1** Inclusion and Exclusion Criteria for the Rapid Review of the Published Literature

| **Inclusion Criteria** | **Exclusion Criteria** |
| --- | --- |
| - Full-text articles | - Articles not available in full-text |
| - Original data | - Non-original data (e.g. systematic reviews) |
| - Peer-reviewed articles | - Grey literature |
| - Any healthcare system/jurisdiction | - Other healthcare professionals |
| - Clinical trials, quasi-experimental studies (including pre-post), observational studies, case studies (including qualitative description) | - Animal studies, commentaries, editorials, letters, opinions |
| - Primary objective: description and/or evaluation of frameworks and/or models for health technology reassessment | - Not focused on primary objective (e.g. lists of candidate technologies for reassessment) |
| - Any outcomes |  |
