# Supplementary material for: Moving low value care lists into action: prioritizing candidate health technologies for reassessment using administrative data
Source: BMC Health Serv Res. 2018 Aug 15;18:640. doi: 10.1186/s12913-018-3459-1 (PMC6094474; doi:10.1186/s12913-018-3459-1)
Supplement: Supplementary file 2 — Appendix 2. Organizations and Agencies Examined for the Environmental Scan. A list of HTA organizations that were searched to identify relevant reports, presentations, guidelines, working papers, or other pertinent grey literature as part of the environmental scan. (DOCX 31 kb) [file 12913_2018_3459_MOESM2_ESM.docx]

**Appendix 2** Organizations and Agencies Examined for the Environmental Scan

| Medical Services Advisory Committee (MSAC - Australia) |
| --- |
| Health Policy Advisory Committee for Technology (HealthPACT - Australia) |
| Pharmaceutical Benefits Advisory Committee (PBAC - Australia) |
| Norwegian Council for Quality Improvement and Priority Setting in Health Care |
| Scottish Health Technologies Group (SHTG) |
| Basque Office for HTA (OSTEBA - Spain) |
| Galician Agency for HTA (Avalia-T - Spain) |
| Swedish Council on Technology Assessment in Health Care (SBU) |
| National Institute for Clinical Effectiveness (NICE - UK) |
| Health Technology Assessment International (HTAi) |
| International Network of Agencies for Health Technology Assessment (INAHTA) |
| International Society for Pharmacoeconomics and Outcomes Research (ISPOR) |
| European Network for Health Technology Assessment (EUnetHTA) |
| International Health Economics Association (IHEA) |
| Agency for Health Research and Quality (AHRQ - USA) |
| Choosing Wisely |
| Canadian Agency for Drugs and Technologies in Health (CADTH) |
| Kaiser International Health Group |
| Blue Cross Blue Shield Association |
| Canadian Provincial Ministries of Health (Quebec, Alberta and Ontario) |
